# Comparison of different surgical approaches for pediatric cataracts: complications and rates of additional surgery during long-term follow-up

**DOI:** 10.6061/clinics/2019/e966

**Published:** 2019-07-22

**Authors:** Camila R Koch, Newton Kara, Marcony R Santhiago, Marta Morales

**Affiliations:** I Departamento de Oftalmologia, Faculdade de Medicina FMUSP, Universidade de Sao Paulo, Sao Paulo, SP, BR; II Sant Joan de Déu Hospital, Barcelona, Spain; III University of Southern California Roski Eye Institute, Los Angeles, CA, USA; IV Departamento de Oftalmologia, Universidade Federal do Rio de Janeiro, Rio de Janeiro, RJ, BR

**Keywords:** Congenital Cataract, Anterior Vitrectomy, Posterior Capsulotomy, Via pars plicata, Via pars plana, Via Corneal

## Abstract

**OBJECTIVES:**

To compare long-term postoperative complications of pediatric cataract surgery with primary intraocular lens (IOL) implantation associated with posterior capsulotomy (PC) and anterior vitrectomy (AV) between patients treated with a corneal or pars plicata/pars plana approach.

**METHODS:**

Children who underwent cataract surgery with in-the-bag primary IOL implantation were divided into two groups according to PC and AV surgical approach: a corneal approach (group 1) and a pars plicata/pars plana approach (group 2). Only patients with a follow-up duration of more than two years were included. Long-term surgical outcomes were retrospectively reported.

**RESULTS:**

The mean follow-up period was 10.00±3.13 years. No cases of glaucoma or retinal detachment were reported. The mean age at surgery was 34.57±22.66 months. Forty-six children were included (27 eyes in group 1 and 29 eyes in group 2). The most frequent postoperative complication was corectopia, followed by visual axis opacification. Both complications occurred more frequently in group 1 (*p*<0.001). After cataract surgery, the rate of additional surgeries in group 1 was 51.9%, while in group 2, the rate was 27.6% (*p*=0.1132).

**CONCLUSION:**

The pars plicata/pars plana approach with PC and vitrectomy with primary in-the-bag IOL implantation for pediatric cataracts is a safe procedure.

## INTRODUCTION

Visual axis opacification (VAO) is the most common complication observed after cataract extraction in children 1,2. In these patients, posterior capsule management is crucial to prevent amblyopia. In cooperative patients, Neodymium:YAG (Nd:YAG) laser capsulotomy can be performed postoperatively when VAO is observed. Otherwise, in young children, a posterior capsulotomy (PC) with anterior vitrectomy (AV) or PC with posterior optic capture with or without vitrectomy should be performed at the time of the cataract surgery 3-6. The following two main approaches are used to perform PC and AV with intraocular lens (IOL) implantation: corneal/limbal and pars plicata/pars plana 7-9.

Advances in phacoemulsification devices, IOL design, surgical techniques and vitrectomy probe technology have made the techniques used in children more reliable 5,10-13. Primary IOL implantation has become more frequent, and research is ongoing to identify better treatments for pediatric cataracts. The literature reports few results comparing surgical approaches, and most of these studies have short-term follow-up periods 14,15. Additional reports are needed to explore the long-term complications in this age group because postoperative complications should be monitored in children throughout life 16,17. In this article, cataract surgery was performed via either a corneal or pars plicata/pars plana approach. Both groups underwent primary PC associated with AV and primary in-the-bag IOL implantation. We report the incidence of postoperative complications and additional surgeries over a long-term follow-up period.

## METHODS

All children were surgically treated for cataracts with primary in-the-bag intraocular lens (IOL) implantation and posterior capsulotomy (PC) with anterior vitrectomy (AV) at the Sant Joan de Déu Hospital from 1997 to 2007. A retrospective review was conducted in accordance with the tenets of the Declaration of Helsinki and was approved by the Medical Institutional Review Board and the Sant Joan de Déu Hospital, Barcelona, Spain. The follow-up period was at least 24 months after the cataract surgery. Patients who underwent PC and AV via the corneal approach were included in group 1, and patients treated via the pars plicata/pars plana approach were included in group 2.

### Exclusion criteria

The preoperative exclusion criteria were systemic anomalies, traumatic cataracts, anterior or posterior segment digenesis, lens subluxation and aniridia. The intraoperative exclusion criteria were posterior capsular rupture, IOL implantation in the ciliary sulcus, IOL implantation with PC but without vitrectomy, the use of PC and AV as secondary procedures, PC and AV via a corneal approach due to a posterior capsular rupture during surgery or surgery performed by another ophthalmologist.

### Surgical techniques

All surgeries were performed by two experienced pediatric cataract surgeons (RP and MM) in patients under general anesthesia with dilated pupils. In both groups, a superior clear 3.2-mm corneal incision was made, and an ophthalmic viscosurgical device (OVD) was injected into the anterior chamber. Then, a manual anterior central continuous curvilinear capsulotomy was performed with capsulorhexis forceps, followed by hydrodissection and lens aspiration. Afterwards, two different surgical approaches were performed.

In the children in group 1, a posterior circular and central manual capsulotomy was performed with capsulorhexis forceps, followed by another corneal clear incision in which an infusion cannula was placed to maintain a balanced salt solution in the anterior chamber during the AV. The vitrectomy probe was inserted into the 3.2-mm incision to perform an AV through the PC site. After IOL implantation, the OVD was aspirated and the incision was sutured with 10-0 nylon.

In the children in group 2, a second corneal clear incision was made after IOL implantation to insert an infusion cannula that allowed a balanced salt solution to be maintained in the anterior chamber. The OVD was aspirated, and primary closure of the corneal incision was performed using 10-0 nylon sutures. The temporal limbal conjunctiva was opened with a pair of scissors, and vessel coagulation was performed. A compass was used to mark the sclera at 2 to 3 mm (depending on the child's age) from the limbus. A central PC and an AV were performed. The sclerotomy site was closed with a single buried 10-0 polyglactin (Vicryl) suture.

In both groups, a 20-gauge vitreous cutting instrument was used at a setting typical for an AV system (i.e., a rate between 500 and 800 cuts per minute and a maximum vacuum of 120 mmHg). An acrylic foldable IOL (MA60BM, AcrySof, Alcon Inc., Fort Worth, TX, USA or Meridian HP60M, Bausch & Lomb, Rochester, NY, USA) was implanted in the bag through the main corneal incision using an injector. At the end of the surgery, an inferior subconjunctival injection of methylprednisolone acetate was administered. Antibiotic drops and a steroid ointment were administered to the operated eye.

### Postoperative care

A topical combination of antibiotic and steroid drops was applied every four hours for one week, and the dose was then tapered over four additional weeks. Oral prednisolone was administered for five days (1.5 mg/kg), and cycloplegic eye drops were used twice daily for two weeks. Follow-up evaluations were performed one day, one week, 30 days, and every three months for up to one year after the surgery, and every six months thereafter.

### Main outcome measures

The main outcome measures were postoperative complications and additional surgeries in groups stratified according to the surgical approach. Glaucoma was defined as an intraocular pressure (IOP) >21 mmHg combined with changes in the cup-to-disc ratio. Two complications were specifically recorded: visual axis opacification (VAO) and corectopia. VAO was defined as lens material regrowth extending into the pupillary space that obscured the visual axis. Corectopia was considered when an irregular pupil was observed. The following data were reviewed: gender, laterality, date of birth, date of surgery, age at surgery, surgeon, surgical technique, type of IOL implanted, number and type of additional surgeries, number and type of complications, date when the complication was first described and follow-up time.

### Statistical analysis

Quantitative variables are presented as means and standard deviations. Qualitative variables are presented as absolute and relative frequencies. A *p-*value <0.05 was considered statistically significant. The Chi-square test was used to identify differences in the frequencies of variables between the surgical techniques. The generalized estimating equation (GEE) was used to compare the means of variables between surgical techniques in bilateral cases. The Statistical Package for the Social Sciences (SPSS Inc. version 19.0, Chicago, USA) for Windows software was used to conduct statistical analyses.

## RESULTS

Fifty-six eyes (46 patients) were included. Thirty-three (58.9%) were associated with bilateral cataracts. The age at the time of surgery was 34.57±22.66 (3 to 108) months, with no difference in the age at operation between groups. The mean follow-up times were 125.21±38.50 (24 to 200) months for all patients, 11.63±3.06 (2 to 16) years in group 1 and 8.48±2.38 (2 to 13) years in group 2. Table 1 shows the demographic and ocular characteristics of the patients stratified by group.

**Figure 1:**
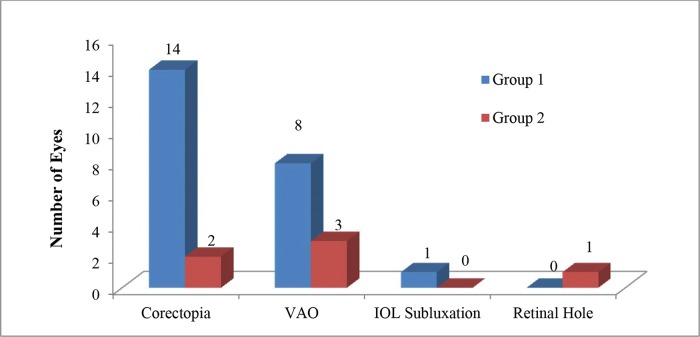
Number of complications in each group. VAO=visual axis opacification; IOL=intraocular lens.

Among the eyes with postoperative complications, 20 (35.7%) had one complication, and nine (16.0%) had two complications. Corectopia was the most frequent complication, followed by visual axis opacification (VAO) and intraocular lens (IOL) subluxation. The mean time after surgery to a diagnosis of corectopia was 1.67±3.35 months and to a diagnosis of VAO was 13.34±14.63 months. One patient with a retinal hole required laser retinal treatment. Four eyes with VAO underwent Neodymium:YAG capsulotomy. No incidences of glaucoma or retinal detachment were reported in either group. Figure 1 shows the number of complications directly related to the surgical procedure in patients stratified by group.

Although an older mean age at surgery was observed in group 1 than in group 2 (*p*<0.0001), a significantly greater number of postoperative complications were observed in group 1 (24, 88.9%) than in group 2 (6, 20.7%) (*p*<0.0001); however, a statistically significant difference in the number of additional surgeries was not observed between groups (*p*=0.112). The number of additional surgeries in each group stratified according to the type of adverse event is shown in Table 2. Additionally, a greater number of postoperative complications occurred in children over the age of two (15 eyes) than in children younger than two years (4 eyes) in group 1, (*p*<0.003), but a similar result was not observed in group 2 (5 eyes in each group) (*p*=0.928).

## DISCUSSION

Although the safely of pediatric cataract surgery has improved, postoperative complications are still common in young children 5 because they are more prone to inflammatory reactions. The maintenance of a clear visual axis is important, particularly during the first 2-3 years of life, when children are more susceptible to amblyopia 18. The posterior capsule must be opened during surgery in young children to avoid this complication, and an anterior vitrectomy (AV) may be performed because the epithelial lens cells can also grow in the anterior vitreous. During the postoperative period in this study, more significant complications, particularly corectopia and visual axis opacification (VAO), were observed when a corneal rather than a pars plicata/pars plana approach was used to perform the PC and the AV; however, no incidences of glaucoma or retinal detachment were observed in either group. We observed better outcomes for patients who received surgery via a pars plicata/pars plana approach over an extensive follow-up period.

The higher vitreous pressure observed in infants might explain why they experience more postoperative complications following surgery using a corneal approach. Using a 23-gauge vitrectomy, Lui et al. 14 also compared different approaches for primary intraocular lens (IOL) implantation in the bag or in the ciliary sulcus. The authors observed greater numbers of intraoperative and postoperative complications, and a greater number of additional surgeries were performed for patients who underwent surgery using the limbal approach. IOL pigmentation was the most frequent complication that occurred during the first month after surgery, followed by VAO.

Most of the intraoperative complications that occurred with the corneal/limbal approach are associated with the iris. Iris trauma during surgery is avoidable if the ability and experience of the surgeon allow the manipulation of the vitrectomy probe in the anterior chamber. In a short follow-up study, Menon et al. 19 described the results associated with a 20-gauge vitrectomy system used with a corneal approach, and no intraoperative corectopia was observed. In our study, no intraoperative corectopia occurred, but it was the most frequent postoperative complication. Recent studies 5,6 have compared IOL optic capture without AV and in-the-bag IOL implantation with AV via a corneal approach. A significant difference in complications was not observed between these groups after one year of follow-up. The technique involving optic capture without AV showed a low rate of intraoperative and postoperative complications, indicating that AV might eventually become an alternative technique; however, additional long-term follow-up studies are needed.

Vitrectomy probes have improved substantially, particularly over the past decade 20. Li et al. 10 reported the short-intermediate-term safety of performing a 23-gauge vitrectomy via a corneal incision in children aged >two years. Currently, a 25-gauge system is used, which achieves satisfactory results for congenital cataracts.^11^,^15^,^21^,^22^ Since smaller-sized instrumentation produces a more stable and deeper anterior chamber, this system may have more advantages than 20- or 23-gauge systems, but a 20-gauge vitrector might be needed in some cases 22. The 25-gauge vitrector was unable to cut the anterior capsule or the fibrous membranes or failed to remove a less-soft lens, even with a maximum cut rate. As shown in the present study, a higher number of complications occurred using a 20-gauge vitrectomy system with the corneal approach. If the decision is made to perform the surgery via a corneal approach, we recommend smaller-sized vitrectomy probes or the IOL optic capture technique without vitrectomy to improve the results.

Although the follow-up periods for the pars plana/pars plicata approach were shorter than for the corneal approach, the follow-up period was considered long. In this study, no cases of glaucoma or retinal detachment were observed during a mean follow-up period of 10±3.1 years. However, the higher rate of intraoperative vitreous disturbance when performing 20-gauge vitrectomy vs 23-gauge or 25-gauge vitrectomy might increase the risk of retinal detachment. Long-term follow-up is required after pediatric cataract surgery because these complications have been reported up to ten years after the surgery 16,17. In our hospital, two visits per year with the pediatric cataract surgeon are routinely required beginning one year after surgery until the child reaches the age of 18 years. Children are also examined by an optometrist who, when necessary, requests an additional visit with the cataract surgeon. If glaucoma or a retinal abnormality is suspected, a visit with a pediatric glaucoma or retina subspecialist is also required.

This study employed the longest follow-up period reported to compare the use of the corneal and pars plicata/pars plana approaches to perform PC and AV for the treatment of pediatric cataracts. In addition, we compared the results obtained using a 20-gauge vitrectomy probe to findings from other studies using 23- or 25-gauge vitrectomy probes. One limitation of this study is its retrospective design. Nevertheless, long-term results were difficult to obtain due to the technological advances in micro-incisions performed with the vitrectomy system.

Even if the corneal approach is considered by anterior segment surgeons as the easiest approach, our short- and long-term satisfactory outcomes for the pars plicata/pars plana approach might influence the future choice of the technical approach 23. In conclusion, the use of a 20-gauge vitrectomy probe with the pars plicata/pars plana approach is safe in children when primary in-the-bag IOL implantation with posterior capsulotomy and vitrectomy is performed to remove pediatric cataracts.

## AUTHOR CONTRIBUTIONS

Koch CR provided substantial contributions to the conception and design of the study, acquisition, analysis and interpretation of data, drafting and critical revision of the manuscript for important intellectual content and approval of final version of the manuscript. Kara-Junior N provided substantial contributions to the conception and design of the study, manuscript drafting and approval of the final version of the manuscript. Santhiago MR provided substantial contributions to the acquisition of data, critical revision of the manuscript for important intellectual content and approval of the final version of the manuscript. Morales M provided substantial contributions to the conception and design of the study, analysis and interpretation of data, drafting and critical revision of the manuscript for important intellectual content and approval of the final version of the manuscript. All authors agreed to be accountable for all aspects of the work to ensure that questions related to the accuracy or integrity of any part of the study are appropriately investigated and resolved.

## Figures and Tables

**Table 1:** Demographic and ocular characteristics of patients stratified by group.

	Group 1	Group 2	*p*-value
Total patients, n (%)	27 (48.2%)	29 (51.8%)	0.217
Laterality, n (uni/bil)	17/10	12/17	0.740
Gender, n (female/male)	12/15	13/16	0.977
Eye, n (OR/OS)	14/13	16/13	0.803
Age at surgery, months (mean±SD)	42.85±25.44	26.86±16.72	<0.0001*
Follow-up period, years (mean±SD)	11.63±3.06	8.48±2.38	<0.0001*

uni=unilateral; bil= bilateral; OR=right eye; OS=left eye; n=number of eyes; SD=standard deviation; *Generalized Estimating Equation (GEE).

**Table 2:** Additional surgeries required postoperatively according to the adverse events that occurred in each group.

	Group 1	Group 2	*p-*value
Additional surgeries	14 (51.9)	8 (27.6)	0.1132*
Surgical treatment, n (%)			
Corectopia	2 (15.4)	1 (12.5)	
VAO	6 (46.2)	1 (12.5)	
IOL dislocation	1 (7.7)	0	
Strabismus	5 (38.5)	5 (62.5)	
Retinal hole	0 (0.0)	1 (12.5)	

* Chi-square test, n=number of eyes; VAO=visual axis opacification; IOL=intraocular lens.
