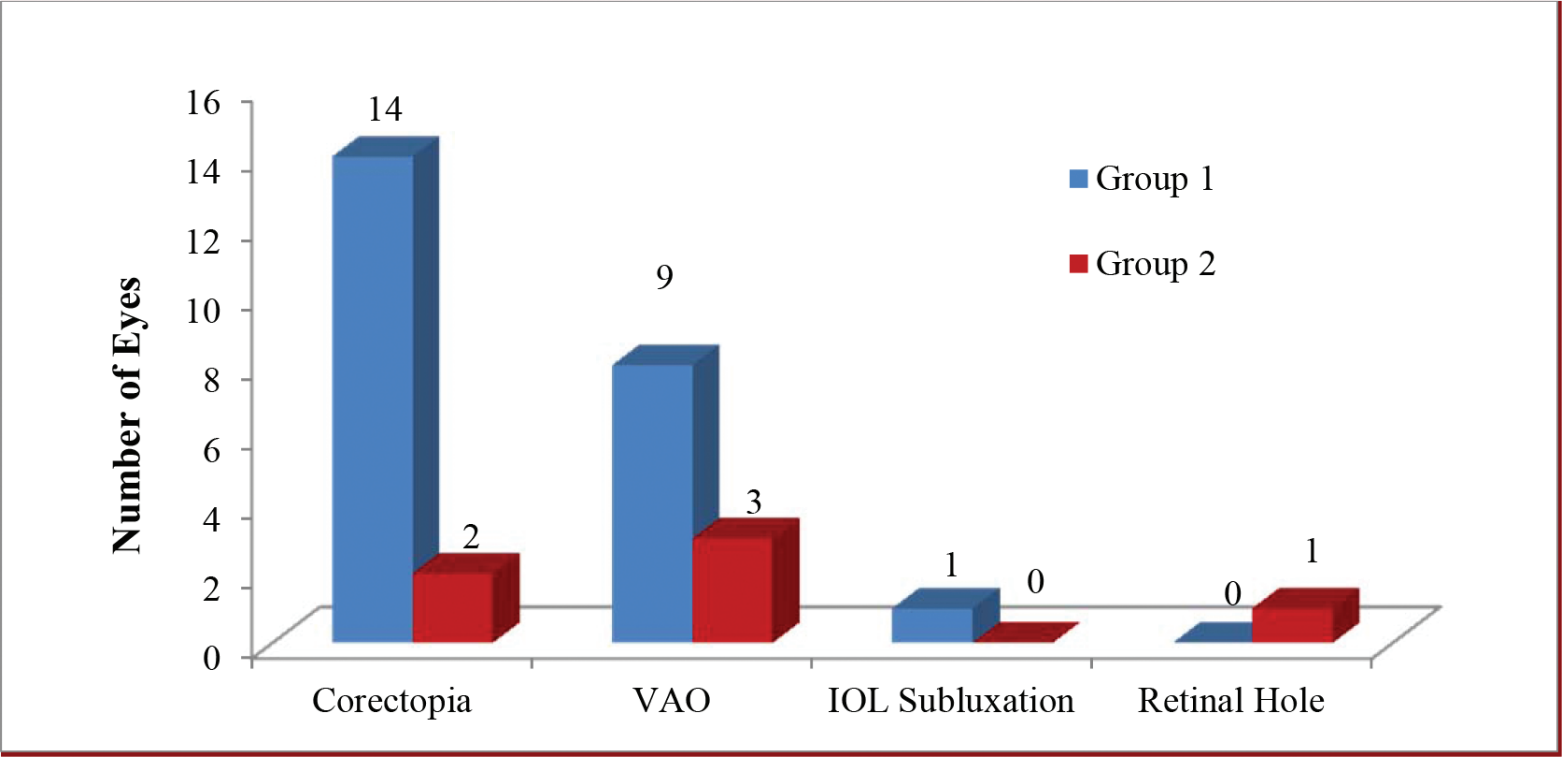# Erratum for: Comparison of different surgical approaches for pediatric cataracts: complications and rates of additional surgery during long-term follow-up

**DOI:** 10.6061/clinics/2020/e966err

**Published:** 2020-04-20

In the article **Comparison of different surgical approaches for pediatric cataracts: complications and rates of additional surgery during long-term follow-up**

Replace **Figure 1** for:

**Figure 1:**